# The Effect of Pluronic-Coated Gold Nanoparticles in Hearing Preservation Following Cochlear Implantation-Pilot Study

**DOI:** 10.3390/audiolres12050047

**Published:** 2022-08-28

**Authors:** Cristina Maria Blebea, Violeta Necula, Monica Potara, Maximilian George Dindelegan, Laszlo Peter Ujvary, Emil Claudiu Botan, Alma Aurelia Maniu, Marcel Cosgarea

**Affiliations:** 1 Department of Otorhinolaryngology, “Iuliu Hatieganu” University of Medicine and Pharmacy, 400337 Cluj-Napoca, Romania; 2 Department of Otolaryngology, Emergency County Hospital, 400006 Cluj-Napoca, Romania; 3 Nanobiophotonics and Laser Microspectroscopy Center, Interdisciplinary Research Institute in Bio-Nano-Sciences, Babes-Bolyai University, T. Laurian Str. 42, 400271 Cluj-Napoca, Romania; 4 Department of Pathology, Emergency County Hospital, 400337 Cluj-Napoca, Romania

**Keywords:** hearing loss, cochlear implant, nanomaterials, ABR, Pluronic, dexamethasone

## Abstract

Introduction: During cochlear implantation, electrode insertion can cause cochlear damage, inflammation, and apoptosis, which can affect the residual hearing. Nanoparticles are increasingly studied as a way to increase the availability of inner ear protective factors. We studied the effect on rats of Pluronic-coated gold nanoparticles (Plu-AuNPs) containing dexamethasone, which were applied locally in the rat’s middle ear following the implant procedure. Methods: Seven rats were used in the study. The right ear served as a model for the Dex-Plu-AuNP group. Following the intracochlear dummy electrode insertion through the round window, Dex-Plu-AuNPs were placed in the round window niche. In the right ear, following the same insertion procedure, free dexamethasone (Dex) was placed in the same manner. Auditory brainstem response thresholds (click stimulus, pure tones at 8 kHz, 16 kHz, 24 kHz, and 32 kHz) were measured before and one week after the procedure. A two-tailed T-test was used for the variables. Statistical significance was set as *p* < 0.05. Results: In the Dex-Plu-AuNP group, the threshold shift was less than that in the free dexamethasone group, but no statistical significance was noted between the groups. When compared individually, only the 8 kHz frequency showed statistically significant, better results after one week, in favor of the Dex-Plu-AuNP group. The mean postoperative 8 kHz threshold in the Dex-Plu-AuNPs was significantly lower than that of the control group (*p* = 0.048, *t*-test). For the other frequencies, statistical analysis showed no significant differences between the mean threshold shifts of the two cohorts. Conclusions: The local application of Plu-AuNPs containing dexamethasone following cochlear implantation may better protect the residual hearing than dexamethasone alone, but a larger sample size is needed to reach a possible statistical significance. Dex-Plu-AuNPs do not seem to cause ototoxicity and may be used as a carrier for other agents. In a clinical setting, Dex-Plu-AuNPs may have the effect of protecting lower frequencies in patients with partial deafness who are candidates for electric acoustic stimulation (EAS). If we consider this tendency, Dex-Plu-AuNPs may also be beneficial for patients with Ménière’s disease.

## 1. Introduction

Reducing cochlear damage and preserving the residual hearing capacity has long been a primary objective of inner ear procedures, particularly cochlear implantation (CI) [1]. In recent years, growing interest can be observed regarding hearing conservation in candidates for hybrid, electro-acoustic stimulation whose residual hearing levels may be affected during CI [2,3]. As patients with partial deafness (PD) can benefit from a CI, members of the HEARRING group have extensively studied hearing preservation in cochlear implantation (HPCI) in adults and children, using a variety of electrode array types and manufacturers [4,5]. Limiting the cochlear trauma during CI is also beneficial for patients with minimal residual hearing as larger amounts of electrically induced information passing through a healthier neural interface will promote better speech discrimination [6].

The loss of hearing remnants following CI surgery, especially for low frequencies, generally presents two possible outcomes: an early and a late phase. The immediate loss of residual hearing can be due to insertion trauma (fracture of the osseous spiral lamina, displacement of the basilar membrane, damage to the stria vascularis, or disturbance of the cochlear fluids) and the subsequent inflammatory response [1]. Later residual hearing loss can occur months after implantation. Such a loss can be progressive, fluctuating, or sudden, and its identification largely depends on the observation period and the frequency of visits to the audiologist [7]. Most of the available information regarding the cochlear inflammatory response and its modulation has been gathered through experimental models; scarce data is available from human postmortem studies. 

The prospects of HPCI have greatly increased with the development of less traumatic electrodes, in conjunction with the application of soft surgery principles [8]. As surgical principles and electrode design have been extensively studied, attention is shifting toward new solutions, such as assisted electrode insertion or otoprotective pharmacological support through either systemic or local drug delivery [1]. Local drug delivery using novel nanoparticles (NPs) via the intratympanic or intracochlear route is intended to generate high localized concentrations while avoiding any systemic side effects. 

Metallic and metal oxide nanoparticles are among the most promising inorganic substances for the treatment of inner ear diseases [9]. Gold nanoparticles (AuNPs) are distinguished by their superior chemical and physical stability. They can be functionalized with organic molecules that are biologically active, granting them excellent biocompatibility, and can be used for loading drugs or as inner ear contrast agents [10,11]. 

NP-based medication delivery can be employed to reduce inflammation and fibrosis after CI and to maintain residual hearing. In recent years, the number of FDA approvals for the use of NPs to treat various auditory diseases has increased [11]. In a preclinical setting, locally applied chitosan-coated gold nanoparticles (Cs-AuNPs) in the rats’ tympanic bulla did not compromise the animals’ hearing thresholds [12]. However, AuNPs have not yet been utilized in treating inner ear diseases. Other types of AuNPs, like pluronic-coated Au-NPs, seem to have promising results regarding drug distribution and hemocompatibility [13]. The current experimental pilot study aims to evaluate if Pluronic-coated AuNPs carrying dexamethasone (Dex-Plu-AuNPs) can yield better auditory brainstem response (ABR) thresholds, compared to free dexamethasone (Dex), and if they can better protect against the progressive cochlear function loss developed following electrode insertion trauma. 

## 2. Materials and Methods

### 2.1. Animals 

Seven adult male Wistar rats, weighing 250–300 g, with good general health were included in a prospective randomized experimental pilot study. Otologic inclusion criteria were considered to be good general health and the absence of otitis media. All the included subjects were endoscopically screened to exclude external and middle ear pathology. All protocols were conducted according to European law regarding the welfare of experimental animals and followed the guidelines established by our institution’s ethical committee (AVZ 81/28.03.2022) and the National Veterinary Health and Food Safety Authority (ethical approval no. 316/30.05.2022). Following the 3Rs approach [14], we minimized the number of subjects by using both ears of each subject to compare the two forms of dexamethasone delivery. 

### 2.2. Chemicals

Trisodium citrate (C_6_H_5_Na_3_O_7_∙2H_2_O, ≥99%), hydrogen tetrachloroaurate (III) hydrate (HAuCl_4_∙3H_2_O, 99.99%), Pluronic F127, and dexamethasone were purchased from Sigma-Aldrich. Ethanol (96%) was obtained from Chemical Company, Iași, Romania. All chemicals were used without further purification. The aqueous solutions were prepared using ultrapure water.

### 2.3. Nanoparticles Preparation and Loading with Dexamethasone 

Citrate-capped gold nanoparticles (AuNPs) of spherical shape were prepared according to the Turkevich–Frens method [15]. In brief, 100 mL of an aqueous solution of HAuCl_4_∙3H_2_O (10^−3^ M) was boiled on a magnetic stirring hot plate. Then, 10 mL of an aqueous sodium citrate solution (38.8 × 10^−3^ M) was quickly added while maintaining magnetic stirring. The stirring process was continued for 10–15 min after the color of the colloidal suspension became a deep burgundy red. The color changes in the solution from yellow to intense burgundy red indicate the formation of colloidal nanoparticles. Pluronic-coated gold nanoparticles (Plu-AuNPs) were obtained by incubating AuNPs with an aqueous Pluronic F127 solution at a final concentration of 4 × 10^−4^ M. The obtained Plu-AuNP suspension was then purified by centrifugation at 12,000 rpm for 20 min. To prepare dexamethasone-loaded Plu-AuNPs (Dex-Plu-AuNPs), the colloidal solution was incubated with Dex at a final concentration of 0.18 mg/mL, and the resulting mixture was ultrasonicated for 50 min. The prepared Dex-AuNP nanoconjugate was then incubated with Pluronic F127 (4 × 10^−4^ M), followed by centrifugation to remove the unbound polymer and drug. The formation of Dex-Plu-AuNPs was monitored by UV–visible absorption spectroscopy, using a Jasco V-670 UV–vis-NIR spectrometer (JASCO, Hachioji, Tokyo, Japan). The concentration of loaded Dex was estimated indirectly, according to the Beer–Lambert law, by calculating the amount of unloaded Dex molecules remaining in the supernatant. The final concentration of loaded Dex was 10 µg/mL. The concentration of gold nanoparticles expressed in µg/mL was determined by atomic absorption spectroscopy (Avanta PM, GBC-Australia (GBC Scientific Equipment, Braeside, Victoria, Australia)).

### 2.4. Anesthesia, Surgical Preparation, and Approach

Anesthesia was achieved using a mix of Ketamine (80 mg/kg) and Xylazine (8 mg/kg), administered intramuscularly [16]. Additional doses were administered during the procedure if signs of pain were observed (withdrawal reflex to a plantar pinch or eyelid reflex). During the procedure, the animal was kept at a constant body temperature of 35–37 °C using a heating pad, and the eyes were protected from drying using a sterile ophthalmic solution. Animals recovered post-operatively in separate cages, with free access to water and food. 

**Figure 1:**
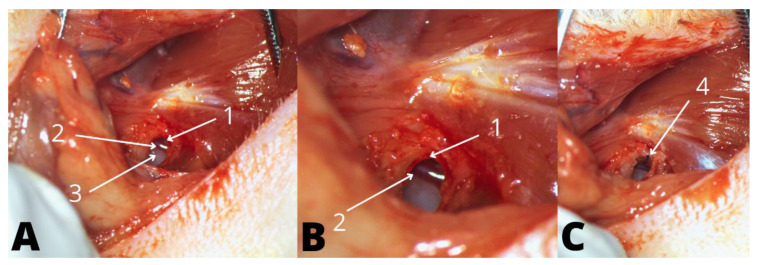
Left ear electrode analog insertion; 1—round window niche; 2—stapedial artery; 3—middle ear, seen through the bony defect; 4—electrode analog during the insertion through the round window membrane. (**A**): Opening of the left middle ear, (**B**): Visualization of the RW, (**C**): Left ear electrode analog insertion.

The surgical intervention was performed using a Leica^®^ microscope (Model M320, Leica, Wetzlar, Hesse, Germany). Insertion of the electrode analog was achieved via the round window (RW), using a retroauricular approach to the tympanic bulla. Access to the middle ear was achieved through a bony defect in the posteroinferior aspect of the bulla. A 1-millimeter diameter cutting drill was used to access the bony defect, with good exposure of the stapedial artery, RW niche, and RW membrane (Figure 1). The bony defect of the middle ear was sealed using the surrounding muscles. Finally, the platysmal muscle and skin were sutured with absorbable Vicryl 6/0 sutures (Ethicon^®^, Raritan, NJ, USA). The wound was covered with a topical spray containing oxytetracycline. All surgical procedures were performed under aseptic conditions.

### 2.5. Dummy Electrode

A monofilament 5/0 polypropylene suture (Ethicon^®^, Raritan, NJ, USA) with a length of 5 mm was employed for the insertion [17,18]. 

### 2.6. Groups

To reduce the number of animals included in the study, we divided the groups as follows. One ear was considered to represent the study group (group 1) with the contralateral ear representing the control group (group 2). In group 1, after inserting the analog electrode through the RW, 30 µL of Dex-Plu-AuNP solution was applied in the middle ear and above the RW. The concentration of Plu-AuNPs was 1005 µg/mL, while the dexamethasone concentration was 64 µg/mL. In group 2, after the same insertion procedure, 30 µL of free Dex solution with a dexamethasone concentration of 64 µg/mL was applied.

### 2.7. Hearing Threshold Measurement: Auditory Brainstem Response (ABR)

All rats included in the study presented normal hearing before surgery. We determined the hearing thresholds bilaterally before surgery and seven days following the implantation procedure. We assessed the external and middle ear status before the audiological measurements, to exclude a conductive hearing loss component.

We measured ABR thresholds in a soundproof room using the Opti-Amp bio amplifier (Intelligent Hearing System, Miami, FL, USA) connected to the Smart EP system. Click stimulus, and pure tones of 8 kHz, 16 kHz, 24 kHz, and 32 kHz were presented through closed tubes placed into the external ear canal, with 1.3 ms duration at a 20/s repetition rate. ER insert earphones were used for the click stimulus and high-frequency transducers were used for the tone burst stimulus. Stainless steel needle electrodes were placed subcutaneously in the retroauricular area (recording negative electrode), on the vertex (positive reference), and rear leg (ground electrode). Ipsilateral evoked potentials were averaged over 1024 sweeps in decreasing intensity increments of 10 dB SPL; thresholds were defined as the minimum stimulating level with an ABR response that can be identifiable and repeatable.

### 2.8. Statistics

IBM^®^ SPSS^®^ (IBM, Armonk, NY, USA) was used for statistical analysis. Analyses were performed on the outcome measures between pre-implantation (T0) and post-implantation (T1). A two-tailed Student’s *t*-test was used for independent variables to assess group heterogeneity, as well as to compare pre- and post-implantation audiological data, represented as dB SPL for the selected frequencies. The significance level was set at *p* < 0.05.

## 3. Results

### 3.1. Spectral Characterization of Dex-Plu-AuNPs

**Figure 2:**
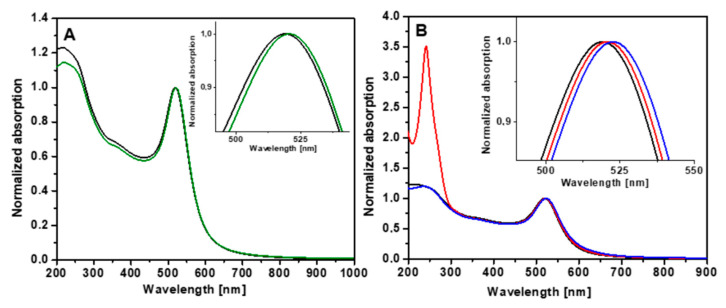
(**A**) Normalized UV-Vis absorption spectra of AuNPs (black) and Plu-AuNPs (green). (**B**) Normalized UV-Vis absorption spectra of AuNPs (black), Dex-AuNPs (red), and Plu-Dex-AuNPs (blue). The inserts show the magnified spectral region of the absorption maxima.

Figure 2A illustrates the normalized UV-Vis absorption spectra of AuNPs before and after stabilization with Pluronic F127. As one can observe, citrate-capped AuNPs exhibit a plasmonic band with an extinction maximum at 519 nm, which represents the typical spectral signature of spherical colloidal gold nanoparticles. Stabilization with Pluronic induced a 2 nm shift toward higher wavelengths, due to the modification of the refractive index of the surrounding medium. Normalized UV-Vis absorption spectra before and after Dex loading are depicted in Figure 2B, taken after each preparation step. The inset of Figure 2B illustrates the magnified spectral region of the absorption maxima. We noticed that the spectra of Dex-AuNPs and Dex-Plu-AuNPs showed a hybrid profile containing both the spectral signature of AuNPs and the electronic absorption of Dex molecules in the UV spectral domain at 240 nm. Moreover, the plasmonic band of Dex-AuNPs experiences a 2 nm red shift compared to AuNPs, followed by a further 2 nm shift toward higher wavelengths upon stabilization with Pluronic. 

These spectral changes are related to the modification of the refractive index of the medium surrounding the particles due to the attachment of Dex onto the gold surface and encapsulation with Pluronic F127. 

### 3.2. Audiological Results

Before cochlear implantation, all rats presented similar hearing thresholds in both ears (Table 1). There was no statistical difference between the median values of the hearing threshold at any of the used stimuli (*p* values > 0.05, *t*-test). 

One week after implantation, neither animal displayed otorrhea, signs of middle ear infection, or infection at the incision site. Therefore, the determinations of ABR thresholds followed the same protocol as the preoperative ones. 

At seven days after bilateral CI, both groups displayed a significant elevation in hearing threshold across all frequency ranges (Table 2). When individual frequencies were analyzed one week after surgery, the Dex-Plu-AuNP group averaged better hearing thresholds than the dexamethasone group. However, no statistical significance was noted between the two groups, except for one frequency. 

At 8 kHz, there was a significant difference of 20.7 dB between the mean thresholds of the two groups in favor of the Dex-Plu-AuNP group (Dex = 108.57, Dex-Plu-AuNP = 87.86, *p* = 0.048, *t*-test) (Figure 2). 

**Figure 3:**
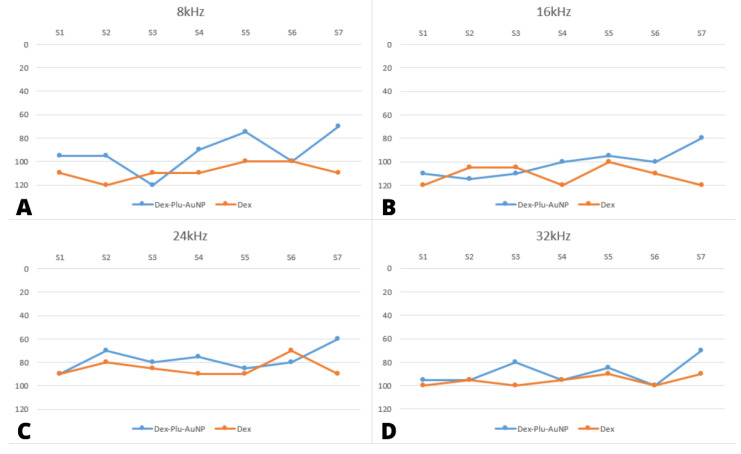
Representation of postoperative hearing threshold for rats in both groups, measured through auditory brainstem response. Results are presented as individual values for each subject at a specific frequency. (**A**) 8 kHz; (**B**) 16 kHz; (**C**) 24 kHz; (**D**) 32 kHz; dB SPL = sound pressure level expressed in decibels; Dex-Plu-AuNP= Pluronic-coated gold nanoparticles with dexamethasone; Dex = free form of dexamethasone; kHz = kilohertz; S = subject.

At 16 kHz, there was a statistically insignificant difference of 11.43 dB between the mean threshold of the two groups in favor of the Dex-Plu-AuNP group (Dex = 108.57 dB, Dex-Plu-AuNPs = 97.14 dB, *p* = 0.21, *t*-test) (Figure 3). At 24 kHz, the difference between the mean threshold of the two cohorts was 8.5 dB; however, this difference did not reach significance (Dex = 79.29, Dex-Plu-AuNPs = 70.71, *p* = 0.32) (Figure 3). In addition, the highest frequency, recorded at 32 kHz, did not yield any significant difference between the groups at 5.7 dB (Dex = 92.86, Dex-Plu-AuNPs = 87.14, *p* = 0.36, *t*-test) (Figure 3). 

**Figure 4:**
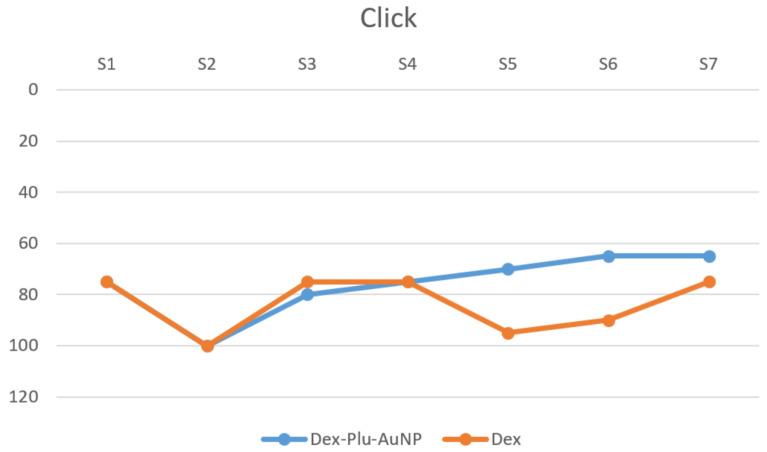
Postoperative hearing threshold using click stimuli; dB SPL = sound pressure level expressed in decibels; Dex-Plu-AuNPs = Pluronic-coated gold nanoparticles with dexamethasone; Dex = free form of dexamethasone; kHz = kilohertz; S = subject.

The click stimulus elicited the least amount of threshold shift in the control and study groups, with a difference of 10.7 dB between them. However, the difference was not statistically significant (Dex = 74.29, Dex-Plu-AuNPs = 63.57, *p* = 0.44, *t*-test) (Figure 4).

## 4. Discussion

Since cochlear surgery can be considered a traumatic event, it is of the utmost importance to maintain the normal architecture and surface anatomy throughout the early periods of recuperation. The reduction of local trauma and preservation of long-term residual hearing are major focuses for patients undergoing cochlear implantation surgery. There have been many attempts to develop adjuvant therapy strategies to avoid or diminish the acute and late inflammatory phases seen after CI. Corticosteroid usage had a substantial influence on auditory brainstem response, impedance, and histopathological alterations in the animal models. However, the otoprotective effect was only long-lasting with continued administration [19,20]. Clinical trials fail to provide consistent recommendations regarding the routes of administration as well as dosage [21,22,23,24]. The cost of hospitalization for intravenous corticosteroid administration and the possible side effects of systemic administration open up the possibility of researching methods of local delivery. To render efficiency and prolong the local effect of corticosteroids, different strategies have been addressed, one of them being the usage of nanocarriers.

AuNPs belong to a promising nanocarrier-mediated drug delivery class; they have easy surface functionalization and the ability to modify their size, shape, and surface chemistry [11]. They also exhibit ROS-independent antimicrobial activity, making them safer for mammalian cells than the other nanometals [25]. Using AuNPs as an intratympanic vector can allow patients to benefit from these characteristics. Nevertheless, the possible variations of these parameters make it hard to truly study their toxicity. Initial in vivo studies researching their utility as an inner ear contrast agent observed no effect on the morphology of the hair cells [12,26]. 

The results of this study confirm some of the previous observations regarding the significant and variable nature of hearing loss after cochlear implantation in experimental models [27]. The limitations of this study would be the reduced number of subjects included. In addition, the nature of the surgical procedure itself can be subject to human variation and needs to be considered a variable.

Our study’s hearing threshold shift was greater than other rodent-based CI experimental models [18]. Although the threshold shift values we obtained in the study were high, another study using the same length (5 mm) of electrode analog also reported high hearing threshold shifts of approximately 65 ± 27 dB [17]. Even though these values are higher than the literature reports, they are consistent throughout all the subjects of both groups; therefore, the repeatability of the surgical maneuvers can be considered to have been accomplished. The full-length insertion of the dummy electrode and its material can be a possible source for the obtained results. The otoendoscopic evaluation eliminated the possibility of middle ear conduction interference that could have influenced the results. 

The results that have emerged from our study show the tendency for a lower hearing threshold shift in the group where CI was associated with Dex-Plu-AuNPs, but no statistical difference was noted except at the 8 kHz frequency. Obtaining better protection of the lower frequencies is the desired effect when considering CI in patients with residual hearing. A larger study group could elicit further information about the sustainability of this finding.

On the other hand, when considering the possible cell toxicity, if no statistical significance was noted between the two groups, it is plausible to add that Plu-AuNPs presented no ototoxicity in the short term. Therefore, this carrier can be considered a safe alternative delivery method for other possible otoprotective agents. In the future, we need to pursue more extended follow-up periods through audiological threshold measures and note histopathological alterations considering different Dex and Plu-AuNP concentrations.

Translating the effect of Dex-Plu-AuNPs in a clinical setting may serve as a better alternative to utilizing free Dex, offering beneficial preventive short-term residual hearing protection from damage caused by the cochlear implantation process itself. The short-term effects do not suggest any cytotoxicity but rather imply a protective effect on hearing thresholds. The potential to better protect lower frequencies needs to be further studied; cochlear implant recommendations have expanded to include patients with partial deafness who are candidates for electric acoustic stimulation (EAS). Clinical implementation can be also beneficial for patients with Ménière’s disease, in which the low frequencies are initially those most affected.

## 5. Conclusions

Pluronic-coated AuNPs carrying dexamethasone, applied to the round window, may be useful as an additional treatment for limiting the short-term inner-ear damage caused by cochlear implantation, along with the soft surgery principles. The long-term effects need to be evaluated, together with the capacity to sustain a delayed and prolonged anti-inflammatory effect. Since no statistical differences were found in the audiological parameters, more evidence is needed to support the use of Plu-AuNPs containing dexamethasone as a pharmacological compound for the protection of residual hearing. Dexamethasone may be beneficial in its free form without the need for a carrier. 

## Figures and Tables

**Table 1:** Preoperative frequency-specific hearing threshold (dB SPL) for both the Dex-Plu-AuNP and Dex groups.

Stimuli	Dex-Plu-AuNPs (*n* = 7)		Dex (*n* = 7)		
	Threshold Mean (dB SPL)	SD	Threshold Mean (dB SPL)	SD	*p* Value (*t*-Test)
Click	20	0	20	0	-
8 kHz	20	0	20.7	1.8	0.35
16 kHz	20	0	20.7	1.8	0.35
24 kHz	20	0	20	0	-
32 kHz	21.4	10.6	22.85	5.6	0.59

The two-tailed *t*-test indicates no significant difference between groups (*p* > 0.05); SD = standard deviation; dB SPL = decibel sound pressure level; kHz = kilohertz; n = number of subjects.

**Table 2:** Postoperative frequency-specific hearing threshold (SPL dB) for both the Dex-Plu-AuNP and Dex cohorts.

Stimuli	Group	N	Mean dB SPL	Std. Deviation	Std. Error Mean
clickpost	Dex-Plu-AuNPs	7	63.57	28.536	10.785
	Dex	7	74.29	21.876	8.268
post8 kHz	Dex-Plu-AuNPs	7	87.86	23.954	9.054
	Dex	7	108.57	6.901	2.608
post16 kHz	Dex-Plu-AuNPs	7	97.14	18.225	6.888
	Dex	7	108.57	14.351	5.424
post24 kHz	Dex-Plu-AuNPs	7	70.71	16.439	6.213
	Dex	7	79.29	14.840	5.609
post32 kHz	Dex-Plu-AuNPs	7	87.14	11.852	4.480
	Dex	7	92.86	10.746	4.062

Dex-Plu-AuNPs = Pluronic-coated gold nanoparticles with dexamethasone; Dex = free form of dexamethasone; dB SPL = decibel sound pressure level; kHz = kilohertz; post = postoperative.

## Data Availability

The data presented in this study are available on request from the corresponding author.
